# Medical Lexicon

**Published:** 1874-02

**Authors:** 


MEDICAL LEXICON: A Dictionary of Medical Science, containing a concise explanation of the various subjects and terms of Anatomy, Physiology, Pathology, Hygiene, Therapeutics, Medical Chemistry, Pharmacology, Pharmacy, Surgery, Obstetrics, Medical Jurisprudence and Dentistry; Notices of Climate and Mineral Waters; Formulæ for Officinal, Empirical and Dietetic preparations, with the accentuation and etymology of the terms and the French and other synonyms: BY ROBLEY DUNGLISON, M.D., L.L.D., late Professor of Institutes of Medicine and Medical Jurisprudence in the Jefferson Medical College of Philadelphia, etc. A new edition enlarged and thoroughly revised by Richard J. Dunglison, M.D., Philadelphia, Henry C. Lea, 1874.

An enlarged edition of the above Lexicon was demanded by the rapid advances made in scientific and medical technical terms. The lamented Prof. Dunglison had begun the work of this Dictionary before his last illness, and the task of completing it devolved upon his son, Dr. Richard J. Dunglison. His mantle fell upon worthy shoulders. Dunglison's Dictionary has been, for forty years, the Medical Lexicon used, generally, by the profession of this county, and they will hail with pleasure the appearance of an edition so comple in every particular as the one before us. "The present edition includes more than *six thousand* subjects and terms not embraced in the last, and although the capacity of the page has been enlarged, the volume has been increased by one hundred pages, so that it contains in fact, additional matter equivalent to at least one hundred and sixty pages of the last edition. Particular care has been devoted to derivation and accentuation of terms. With regard to the latter, indeed, the present edition may be considered a complete Pronouncing Dictionary of Medical Science." It is perhaps the most reliable work published for the busy practitioner, as it contains information upon every medical subject—in a form for ready access and with a brevity as admirable as it is practical. Every Doctor should, by all means, place the late edition of this Dictionary among the best and choicest selections for his library.

